# Tillage, green manure and residue retention improves aggregate-associated phosphorus fractions under rice–wheat cropping

**DOI:** 10.1038/s41598-022-11106-x

**Published:** 2022-05-03

**Authors:** Sandeep Sharma, Sukhjinder Kaur, Om Parkash Choudhary, Manpreet Singh, Asma A. Al-Huqail, Hayssam M. Ali, Ritesh Kumar, Manzer H. Siddiqui

**Affiliations:** 1 Department of Soil Science, Punjab Agricultural University, Ludhiana, 141004 India; 2 Department of Farm Machinery and Power Engineering, Punjab Agricultural University, Ludhiana, Punjab India; 3 Department of Botany and Microbiology, College of Science, King Saud University, Riyadh, 11451 Saudi Arabia; 4 Department of Agronomy, Kansas State University, Manhattan, KS 66506 USA

**Keywords:** Biochemistry, Microbiology

## Abstract

The sustainability of the rice–wheat system is threatened due to the deterioration of soil health and emergence of new challenges of climate change caused by low nutrient use efficiency and large scale burning of crop residues. The conservation agriculture based on tillage intensity, crop residue retention and raising green manuring (GM) crops during the intervening period between wheat harvest and rice establishment offers opportunities for restoration of phosphorus (P) dynamics and stimulate phosphatase activities within the macro-and micro-aggregates. Phosphorus and phosphatase activities in the soil aggregates affected by different residue management practices remain poorly understood. Thus, soil samples were obtained after a five-year field experiment to identify the effect of tillage, green manure and residue management on aggregate-associated phosphorus fractions. Four main plot treatments in rice included combination of wheat straw and GM were conventional till puddled transplanted rice (PTR) with no wheat straw (PTR_W0_), PTR with 25% wheat stubbles retained (PTR_W25_), PTR without wheat straw and GM (PTR_W0_ + GM), and PTR with wheat stubbles and GM (PTR_W25_ + GM). Three sub-plots treatments in the successive wheat crop were conventional tillage (CT) with rice straw removed (CTW_R0_), zero tillage (ZT) with rice straw removed (ZTW_R0_) and ZT with rice straw retained as surface mulch (ZTW_R100_). Results of the present study revealed significantly higher phosphorus fractions (HCl-P, NaHCO_3_-P_i_ and NaOH-P_o_) in treatment PTRW_25_ + GM and ZTW_R100_ compared with PTRW_0_/CTW_R0_ within both macro- and micro-aggregates. The total phosphorus (P), available P, alkaline phosphatase and phytin-P were significantly higher under ZTW_R100_ than CTW_R0_. The principal component analysis identified NaOH-P_o_, NaHCO_3_-P_i_ and HCl-P as the dominant and reliable indicators for evaluating P transformation within aggregates under conservation agriculture-based practices.

## Introduction

Soils play an essential part in maintaining agroecosystem productivity and understanding the impacts of management practices for agricultural sustainability^1,2^. Tillage and residue management practices resulted in improves soil structure, associated protection of SOM and biological activities^3–5^, which eventually improves organic matter, soil aggregation, and nutrient cycling in agricultural systems^6–8^. Phosphorus (P), being the second most imperative nutrient, remained less available to plants due to its adsorption and precipitation with iron, aluminum and calcium in soils, thereby resulting in the rapid formation of non-labile P forms^9,10^. Soil P transformations vary depending on the soil type and management practices^11^. Moreover, the continuous application of phosphate fertilizers has led to serious environmental threats including acidification, hardening and P leaching from the soil^12^. The sustainability of conventional RWS based on intensive tillage is threatened by the scarcity of water, energy and labour, higher production cost and environmental pollution due to the burning of crop residues and deteriorating soil health^7,13,14^ and emerging challenges associate with climate change^15,16^. To address the aforementioned issues, CA-based practices (minimum tillage, residue retention and crop diversification) are being developed and promoted for rice and wheat production^17^. The alternative systems of tillage, GM and residue management practices under CA-based practices can potentially lead to significant changes in the availability of nutrients in RWS^8,18^. The CA-based practices hold the potential to enhance P availability by altering the soil microbial diversity and enzyme activity, which in turn affects the availability of soil P^19–21^. These practices can regulate the accumulation and depletion of the soil organic matter (SOM), carbon (C) sequestration, soil aggregation through microbial processes^22^ and contribute to higher crop yields^23^.

Soil aggregate size, distribution and stability perform an important role in enhancing the physico-chemical and biological processes in soil^24^ and also affects the P forms and availability^25^. The organic phosphorus (P_o_) is usually found in chemically or physically protected forms, which are mineralized slowly into available forms for plant uptake, mostly as a byproduct of SOM decomposition or through the action of specific enzymes. The constant loss of P reservoir in the soil owing to crop harvesting, runoff and leaching can also consume P_o_ and P_i_ forms rapidly, and thus resulting in the deficiency of P to plant. This P loss is directly associated with the stability of soil aggregates and nutrients distribution within aggregates^26^. The P distribution among various fractions indicates the potential stability of P in soil and may vary with different management practices^27^. In addition, stable aggregates reduce soil erosion and degradation, surface runoff and crusting^22^. These CA-based practices comprising ZT with crop residues retention enhances soil aggregation, mean weight diameter of water-stable aggregates and also increase the resistance of aggregates to slaking^3,28^. We hypothesized that tillage, GM and residue management practices may lead to significant changes in P-fractions with in soil aggregates. Furthermore, the focus was to elucidate the distribution of different fractions of P (P_i_ and P_o_) in the aggregates and how these fractions affected the crop yield under CA-based RWS.

## Materials and methods

### Site description

A field experiment (5-year) on irrigated RWS was initiated in 2011 with rice crop on a sandy loam soil classified as *Typic Ustochrept* (USDA classification) at the research farm of the Punjab Agricultural University, Ludhiana, Punjab (30°56′N, 75°52′E, 247 m ASL) Punjab, India.

The electrical conductivity, pH (1:2 soil: water), oxidizable carbon (SOC)^29^, available-P^30^ and available-K^31^ content of 0–15 cm layer of soil was 0.34 dS m^−1^, 7.81, 3.51 g kg^−1^_,_11.3 mg P kg^−1^_,_ 46.3 mg K kg^−1^ , respectively as explained in Saikia et al.^5^. The region is characterized by a sub-tropical semi-arid type of climate with a hot summer (March-June), wet monsoon season (late June-mid September) and a very cold winter (October-February). There were four main plot treatment combinations of wheat straw and *Sesbania aculeate* green manure management in rice (PTR_W0,_ puddled transplanted rice with no wheat straw; PTR_W25_, puddled transplanted rice with 25% anchored wheat stubbles retained; PTR_W0_ plus GM, and PTR_W25_ plus GM). The treatments in the sub-plots consisted of three combinations of tillage and residue management in subsequent wheat (CTW_R0_, conventionally tilled wheat with rice residue removed; ZTW_R0_, zero tilled wheat with rice residue removed and ZTW_R100_, zero tilled wheat with 100% rice residue retained as mulch). The GM at the age of 6–7 weeks was incorporated into the soil in the second week of June by two disking followed by two harrowing and planking before transplanting of rice. The amount of dry matter of GM and rice straw was added ranged from 3.46 to 4.1 Mg ha^-1^ and from 8.15 to 8.97 Mg ha^-1^, respectively in different years under different treatments. The complete detail of experimental information is provided in Saikia et al.^5^ and only details relevant to the present study are discussed here (Table 1).

**Table 1:** Description of treatments.

Abbreviation	Treatment detail	Method of crop establishment
**Rice (main plot treatments)**		
PTR_W0_	Conventional tillage with puddled transplanted rice (PTR) + wheat straw removed (W_0_)	Residue of preceding wheat was removed. Pre- puddling tillage operations included two discings and two harrowings followed by plankings. Wet tillage (puddling) was done twice in 6–8 cm of standing water using a tractor-mounted puddler followed by planking. Rice seedlings were manually transplanted at 15 × 20 cm spacing
PTR_W25_	PTR with wheat straw retention (W_25_)	Anchored (10–12 cm high) wheat straw (25%) of preceding wheat was retained in the field. All the tillage and rice crop establishment operations were same as in PTR_W0_
PTR_W0_ + green manure (GM)	PTR with GM	Residue of preceding wheat was removed and *Sesbania aculeate* (dhaincha) green manure was sown after wheat harvest with zero tillage (ZT). Green manuring was done after 6–8 weeks of sowing, chopped with two passes of disc harrows and incorporated into soil with two passes of cultivators Puddling and rice establishment operations were same as in PTR_W0_
PTR_W25_ + GM	PTR with wheat straw retention + green manure	Anchored (~ 10–12 cm high) wheat residue (25%) of preceding crop was retained in the field. *Sesbania aculeate* green manure incorporation and puddling operations were same as in PTR_W0_ + GM. Rice seedlings were manually transplanted at 15 × 20 cm spacing
**Wheat (sub-plot treatments)**		
CTW_R0_	Conventional till wheat with rice straw removal	All the residue of previous rice crop was removed. Tillage operations included two passes of harrows and two passes of tine plough followed by plankings. After pre-sowing irrigation, seed bed was prepared by two passes of tine plough followed by planking. Wheat was sown using seed cum fertilizer drill in rows 20 cm apart
ZTW_R0_	Zero till wheat with rice straw removal	Residue of previous rice crop was removed. Wheat was direct seeded in the no till plots in rows 20 cm apart using zero till seed-cum-fertilizer drill
ZTW_R100_	Zero till wheat with rice straw retention	Residue of previous rice crop was retained. Wheat was direct seeded in rows 20 cm apart into rice residues using Turbo Happy Seeder

### Soil analysis

Undisturbed soil clods measuring about 50 cm in diameter from the soil layer (0–15 cm) were collected in April-2016 from each plot in duplicate after wheat harvest (after five years of RWS) using hand shovels for analysis of the aggregate size and different fractions of P. After shade drying, soil clods were left to fall from waist height on to a grassy surface to naturally break at of cleavage.

### Soil physical characteristics

For aggregate analysis, the samples were passed through a 4.0 mm sieve. The aggregates retained over a 2.0 mm sieve were retained for aggregate size analysis. A nest of six sieves (2.0, 1.0, 0.50, 0.25, 0.11 and 0.053 mm) were used for wet sieving of aggregate^32^ and weighed each sieve after drying. The aggregate samples were collected from sieves 2 mm, 1–2 mm, below 0.25 mm for micro-aggregates and above 0.25 mm sieves for macro-aggregates and above using the sequential extraction procedure as given by Sui et al.^33^ was employed for P fractionation studies. Total P in the samples was analyzed by the method given by Alexander and Robertson^34^. Available P was estimated using the methods described by Olsen et al.^30^ by using 0.5 N NaHCO_3_ (pH 8.5) as extracting agent. The intensity of blue color was directly proportional to the P content in soil and was read on a colorimeter at a wavelength of 660 nm. The alkaline phosphatase (Alk-P EC 3.1.3.1) activity was assayed based on p-nitrophenol (pNP) release after cleavage of enzyme-specific synthetic substrates^35^. This is based on the colorimetric estimation of the p-nitrophenol released when soil is incubated with buffered (pH 11) sodium p-nitrophenyl phosphate solution. The phytin P was estimated by extraction of phytate with 15% CCl_3_-COOH (trichloro-acetic acid) as described by Mega^36^.

### Statistical analysis

The data were statistically analyzed using analysis of variance technique in split plot design using CPCS1, locally developed software^37^. Mean separation for different treatments was performed using the Least Significant Difference (LSD) test. Differences in treatments for dif *p* < 0.05 were considered statistically significant. The principal component analysis (PCA) method is a statistical tool to avoid any biasness^38^. The soil quality index (SQI) was calculated using the integrated score and weight factor of each indicator using equation^39^.

$${\text{Soil quality index }}\left( {{\text{SQI}}} \right) \, = \sum\nolimits_{{{\text{i}} = {1}}}^{{\text{n}}} {{\text{W}}_{{\text{i}}} {\text{X S}}_{{\text{i}}} }$$

where, ‘S’ = indicator score and W = PCs weight factor.

## Results

### Aggregate-associated inorganic and organic phosphorus fractions

**Figure 1:**
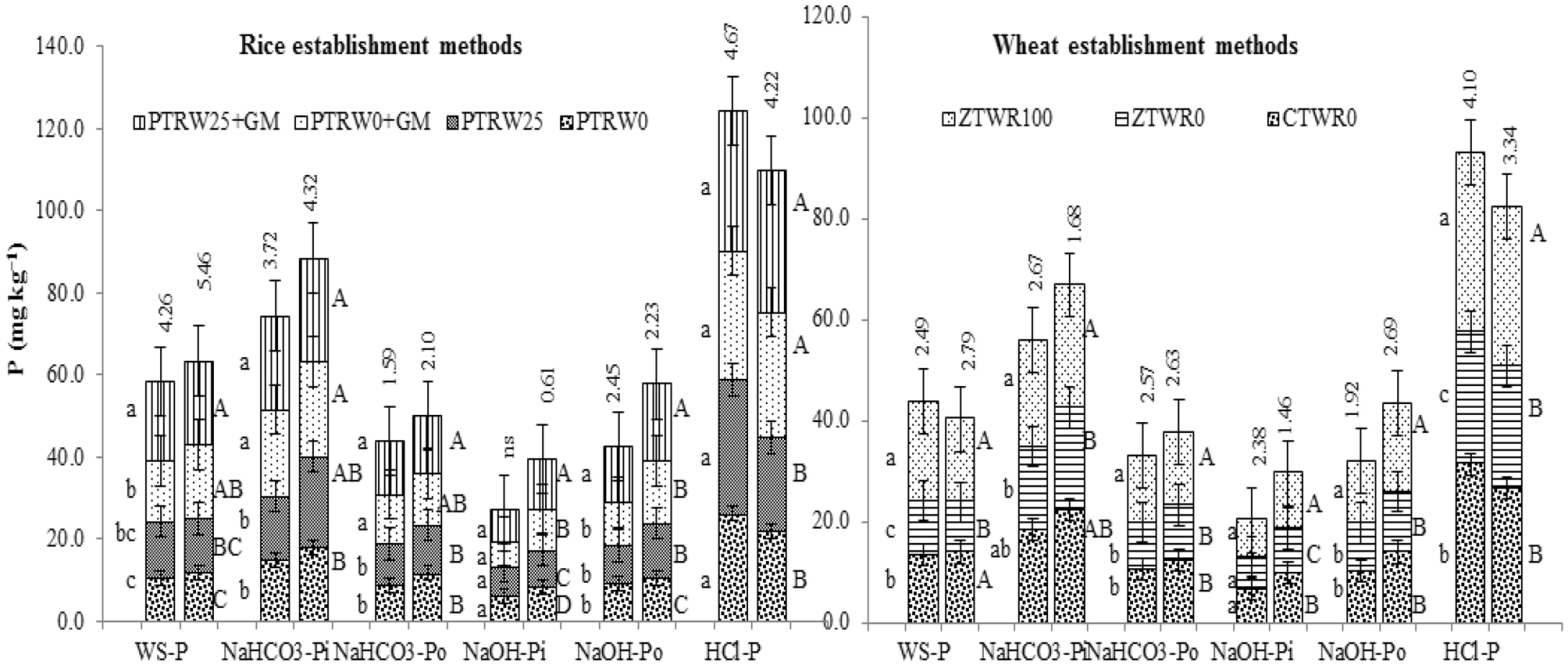
Effect of tillage, green manure and residue management practices on phosphorus fractions in micro-aggregate (< 0.25 mm) and macro-aggregate (> 0.25 mm) after five years of rice–wheat cropping system. PTR_W0_-puddled transplanted rice with no wheat straw, PTR_W25_-puddled transplanted rice with 25% anchored wheat straw retained, GM-Green manure, CTW_R0_-conventional tillage wheat with rice straw removed, ZTW_R0_-zero tillage wheat with rice straw removed, ZTW_R100_-ZTW with rice straw retained as surface mulch; Vertical bars are the standard errors of the mean (*p* < *0.05*). First and second stacked bars indicate micro-aggregate and macro-aggregate, respectively. The values above the vertical bars represent least significant difference test.

**Figure 2:**
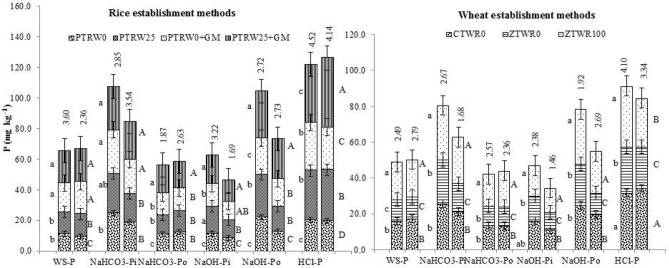
Effect of tillage, green manure and residue management practices on phosphorus fractions in micro-aggregate (1–2 mm) and macro-aggregate (> 2 mm) after five years of rice–wheat cropping system. PTR_W0_-puddled transplanted rice with no wheat straw, PTR_W25_-puddled transplanted rice with 25% anchored wheat straw retained, GM-Green manure, CTW_R0_-conventional tillage wheat with rice straw removed, ZTW_R0_-zero tillage wheat with rice straw removed, ZTW_R100_-ZTW with rice straw retained as surface mulch. Vertical bars are the standard errors of the mean (*p* < *0.05*). First and second stacked bars indicate 1–2 mm and > 2 mm, respectively. The values above the vertical bars represent least significant difference test.

The tillage, GM and residue management practices in the present study had posed significant effects on the P fractions across different sized aggregates (Figs. 1, 2). In the micro-aggregates fraction (< 0.25 mm), the water-soluble phosphorus (WS-P) in PTR_W25_ + GM was significantly 29.2, 39.0, and 82.9% higher than PTR_W0_ + GM, PTR_W25_ and PTR_W0,_ respectively_._ Likewise, for other P fractions, PTR_W25_ + GM recorded significantly higher NaHCO_3_-P_i_ than PTR_W0_ + GM, PTR_W25_ and PTR_W0_ by 8, 46, 52% for NaHCO_3_-P_i_; NaHCO_3_-P_o_ by 7.4, 27.5, 49.4% and NaOH-P_i_ by 22.5, 5.5, 22.5%, respectively. In contrast to the above fractions, the significantly highest levels of NaOH extractable P_o_ (13.6 mg kg^-1^) were obtained in treatment PTR_W0_ + GM. Moreover, the HCl-P was the dominant P-fraction, followed by NaHCO_3_-P_i_. In contrast to other P-fractions, the maximum HCl-P in aggregate size < 0.25 mm and > 0.25 mm was recorded in treatment PTR_W25_ + GM followed by PTR_W0_ + GM, respectively. Similar results were observed for the relative distribution of P under aggregate size 1–2 and > 2 mm (Fig. 2).

Among tillage and residue retention practices in wheat, ZTW_R100_ resulted in a significantly higher P concentration in all the inorganic and organic P-fractions (WS-P, NaHCO_3_-P_o_, NaHCO_3_-P_i_, NaOH-P_o_, NaOH-P_i_ and HCl-P) across different aggregate sizes compared with ZTW_R0_ and CTW_R0_ (Figs. 1  and 2)_._ The HCl-P was found to be the dominant fraction which increased significantly in treatment ZTW_R100_ by 13.3 and 35.9%; 18.4 and 30.2%; 7.3 and 32.8% and 25.7 and 47.5% in < 0.25 mm, > 0.25 mm, 1–2 mm and > 2 mm size aggregates compared with ZTW_R0_ and CTW_R0,_ respectively. Hence, the comparison of various P fractions within different aggregate size classes revealed a significantly higher concentration of P in PTR_W25_ + GM and ZTW_R100_.

### Soil total, available and alkaline phosphatase

**Figure 3:**
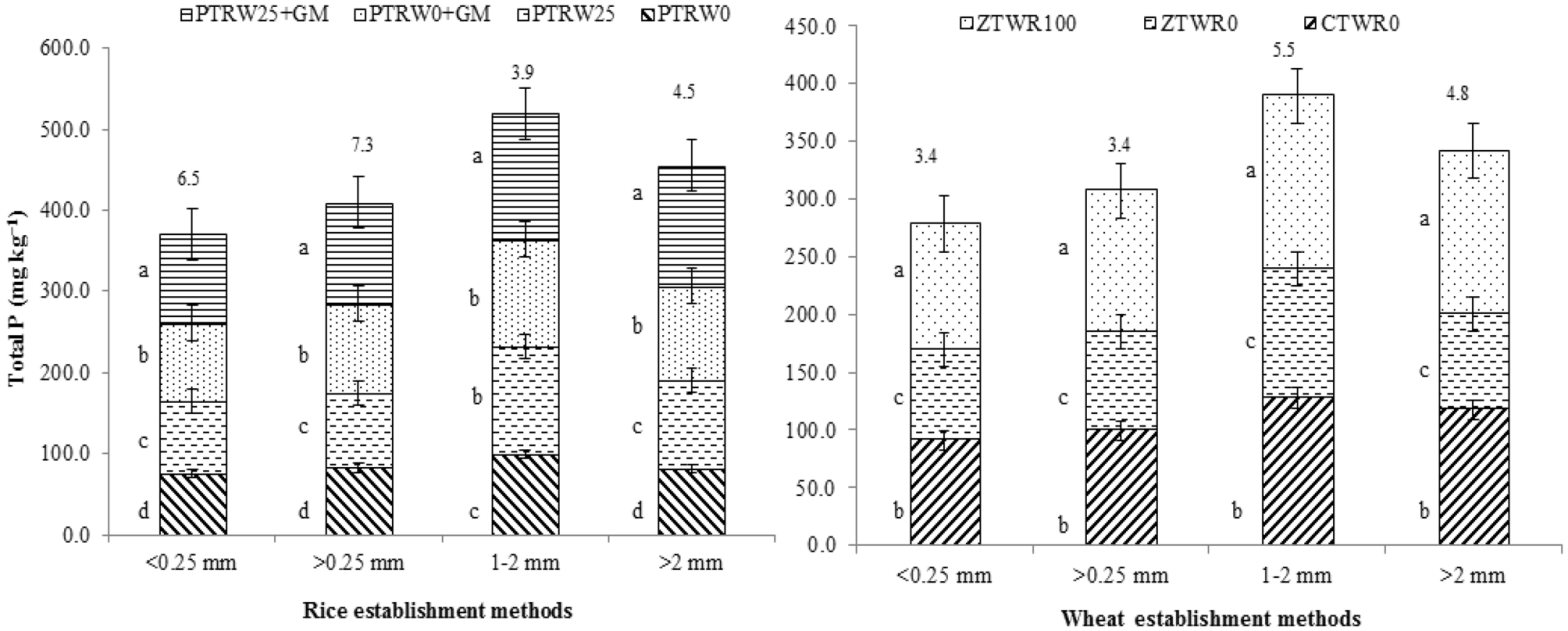
Effect of tillage, green manure and residue management practices on total phosphorus within aggregates after five years of rice–wheat cropping system. PTR_W0_-puddled transplanted rice with no wheat straw, PTR_W25_-puddled transplanted rice with 25% anchored wheat straw retained, GM-Green manure, CTW_R0_-conventional tillage wheat with rice straw removed, ZTW_R0_-zero tillage wheat with rice straw removed, ZTW_R100_-ZTW with rice straw retained as surface mulch. Vertical bars are the standard errors of the mean (*p* < *0.05*). The values above the vertical bars represent least significant difference test.

The distribution of P_i_ and P_o_ fractions within different aggregate classes showed maximum total P in the aggregate size 1–2 mm followed by > 2 mm then > 0.25 mm (macro-aggregates) and least in the < 0.25 mm (micro-aggregates) (Fig. 3). The average total P ranged from 84.8 to 135.0 mg kg^−1^ with the highest observed in the treatment PTR_W25_ + GM followed by PTR_W0_ + GM, PTR_W25_ and PTR_W0._ Compared to the PTR_W0_ and PTR_W25_ treatments, the PTR_W25_ + GM increased the soil total P by 59.2% and 27.7%, respectively, in the fraction 1–2 mm. Thus, the total P in aggregate fraction 1–2 mm accounted for the major P proportion (130 mg kg^−1^), consistent with the overall distribution of aggregates under various treatments. Among the tillage and residue management practices in wheat, ZTW_R100_ accumulates the significantly higher total P over ZTW_R0_ and CTW_R0_, in macro- as well as micro-aggregates. Here also, the aggregate size 1–2 mm possessed the maximum total P compared to other aggregate size particles.

**Figure 4:**
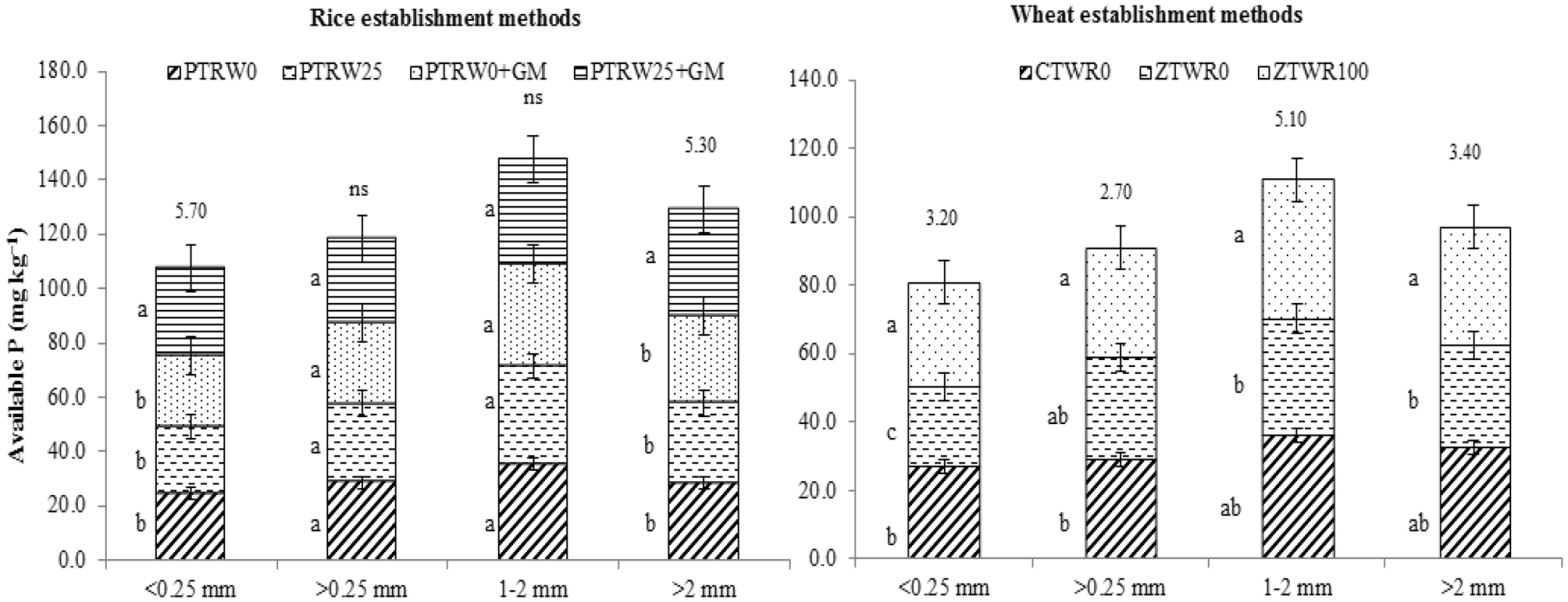
Effect of tillage, green manure and residue management practices on available phosphorus within aggregates after five years of rice–wheat cropping system. PTR_W0_-puddled transplanted rice with no wheat straw, PTR_W25_-puddled transplanted rice with 25% anchored wheat straw retained, GM-Green manure, CTW_R0_-conventional tillage wheat with rice straw removed, ZTW_R0_-zero tillage wheat with rice straw removed, ZTW_R100_-ZTW with rice straw retained as surface mulch Vertical bars are the standard errors of the mean (*p* < *0.05*). The values above the vertical bars represent least significant difference test.

The available-P was also highest in aggregate size, 1–2 mm followed by > 2 mm then > 0.25 mm while least was observed in < 0.25 mm (Fig. 4). The average available-P was recorded to be the significantly higher in PTR_W25_ + GM by 12.2%, 18.0% and 20.4% compared with PTR_W0_ + GM, PTR_W25,_ and PTR_W0_, respectively. Consistent with the results of total P, the ZTW_R100_ also exhibited significantly higher available P by 32.5 and 13.3% in < 0.25 mm, 7.4 and 10% in the > 0.25 mm, 18.7 and 13.1% in the 1–2 mm and 15.3 and 5.8% in the > 2 mm over ZTW_R0_ and CTW_R0_, respectively.

**Figure 5:**
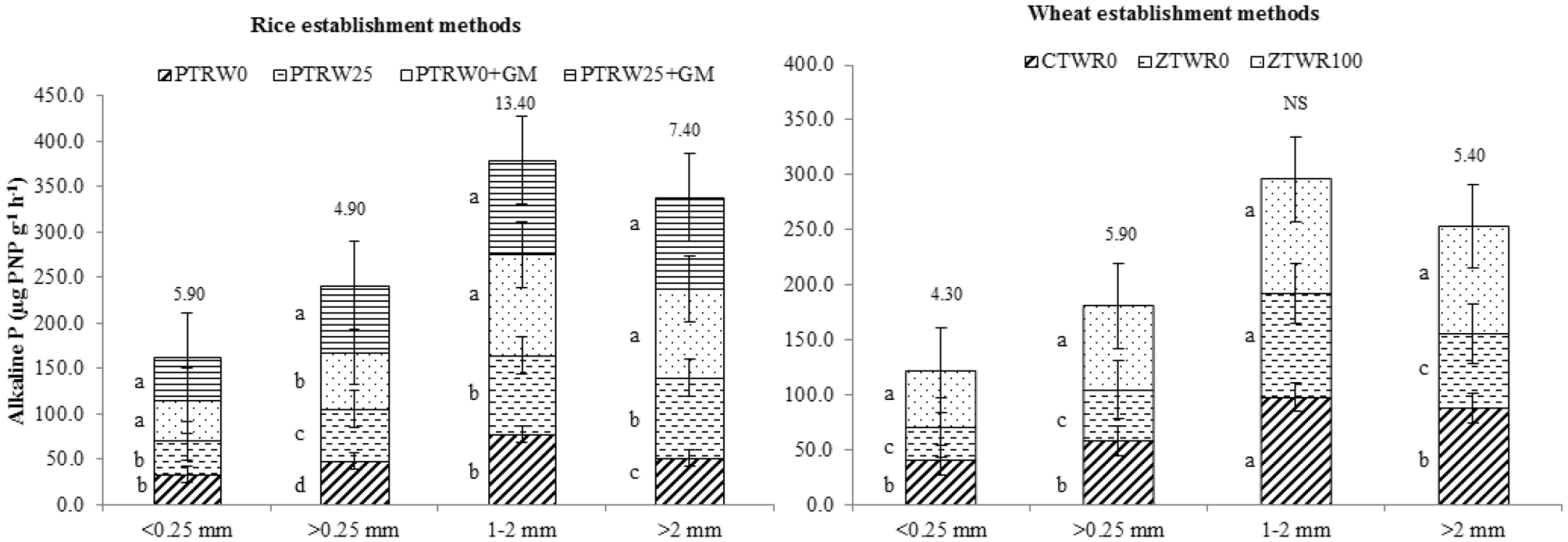
Effect of tillage, green manure and residue management practices on alkaline phosphatase within aggregates after five years of rice–wheat cropping system. PTR_W0_-puddled transplanted rice with no wheat straw, PTR_W25_-puddled transplanted rice with 25% anchored wheat straw retained, GM-Green manure, CTW_R0_-conventional tillage wheat with rice straw removed, ZTW_R0_-zero tillage wheat with rice straw removed, ZTW_R100_-ZTW with rice straw retained as surface mulch. Vertical bars are the standard errors of the mean (*p* < *0.05*). The values above the vertical bars represent least significant difference test.

**Figure 6:**
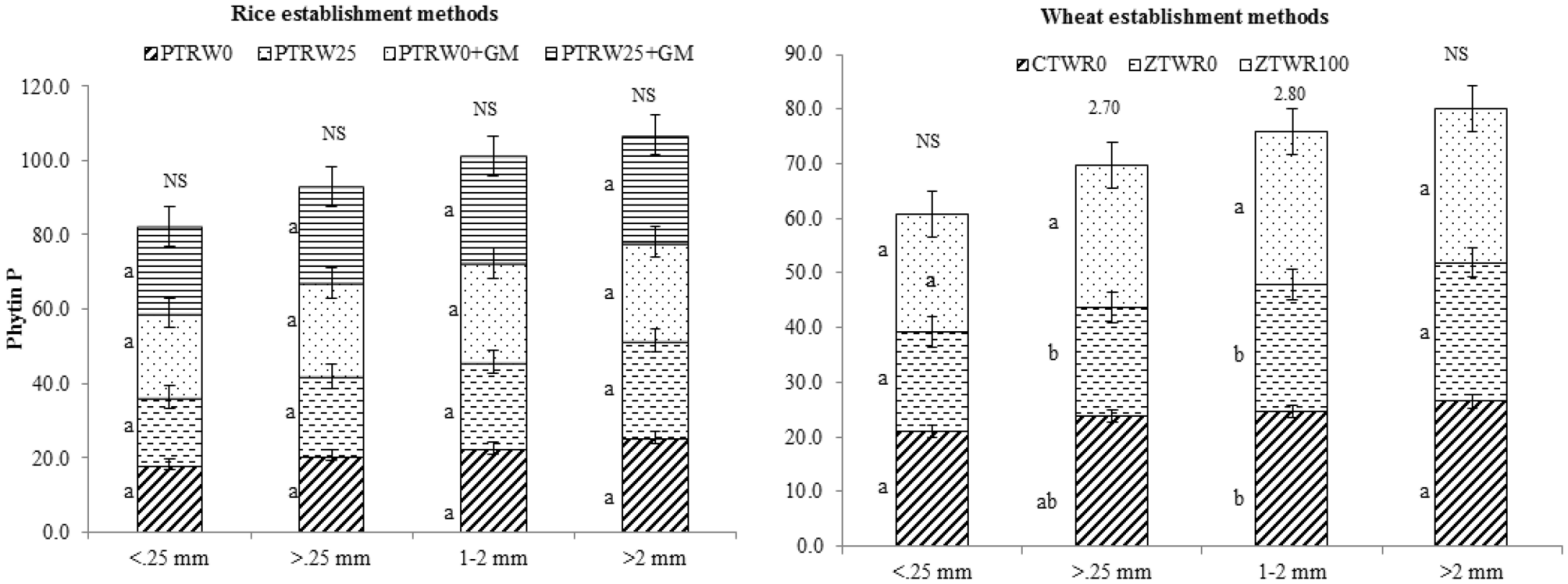
Effect of tillage, green manure and residue management practices on phytin-P within aggregates after five years of rice–wheat cropping system. PTR_W0_-puddled transplanted rice with no wheat straw, PTR_W25_-puddled transplanted rice with 25% anchored wheat straw retained, GM-Green manure, CTW_R0_-conventional tillage wheat with rice straw removed, ZTW_R0_-zero tillage wheat with rice straw removed, ZTW_R100_-ZTW with rice straw retained as surface mulch. Vertical bars are the standard errors of the mean (*p* < *0.05*). The values above the vertical bars represent least significant difference test.

Among rice treatments, higher alkaline phosphatase activity and phytin-P content were observed in the treatment PTR_W25_ + GM which were 2.5 and 4.2% higher than PTR_W0_ + GM_,_ 20.6 and 20.7% than PTR_W25_ and 54.1 and 22.6% than PTR_W0,_ respectively (Figs. 5 and 6). In both the aggregate fractions (< 0.25 mm and > 0.25 mm), significantly higher alkaline phosphatase activity (*p* < 0.05) and phytin-P (*p* < 0.05) content were recorded in treatment PTR_W25_ + GM. The macro-aggregate fraction contributed higher towards the enzyme activity and phytin-P content than micro-aggregate. In wheat treatments, when 100% rice residue was incorporated along with GM in ZT wheat, the alkaline phosphatase activity and phytin-P content were highest irrespective of the type of fraction (Fig. 6). The average activity varied from 59.3 to 82.0 µg pNPg^-1^ h^-1^ and 25.98 to 21.65 mg kg^-1^ in the ZTW_R100_, ZTW_R0_ and CTW_R0_, respectively. Thus, our results indicated that among the different sized fractions, 1–2 mm possessed maximum available P, total P, alkaline phosphatase activity and phytin-P content compared with other aggregate size particles.

### Crop yield and nutrient uptake

Conservation agriculture-based practices in rice significantly affected the grain yield (Table 2). The grain yield in PTR_W25_ + GM was increased by 11%, 15% and 26% compared with PTR_W0_ + GM, PTR_W25_ and PTR_W0,_ respectively. Similarly, the tillage-based crop establishment treatments in wheat significantly affected the grain yield, straw yield, grain and total P uptake (Table 3). The increase in grain yield under ZT along with residue retention was 19% and 9% higher than ZT and CT without residue, respectively. Like grain yield, rice residue management practices showed a higher effect on grain yield attributes. The grains spike^-1^ was significantly higher under ZTW_R100_ than that of ZTW_R0_. Grain yields of wheat were significantly related to total P and NaOH-P_o_ fraction (r = 0.997** and 0.988*, respectively, *p* < 0.05) (Table 4). The available P and alkaline phosphatase activity also exhibited significant relationships with the wheat grain (r = 0.968** and 0.949**, respectively, *p* < 0.05).

**Table 2:** Effect of tillage, green manure and residue management practices on yield and yield attributes of wheat.

Treatment	Grain yield	Straw yield	Harvest Index	1000 grain weight	Spike length (cm)	No of grains/ spike
	(t ha^-1^)					
**Rice establishment methods**						
PTR_WO_	4.71b	6.69	0.412	31.2	12.7	64.7
PTR_W25_	5.15b	6.8	0.429	31.1	12.4	67.8
PTR_WO_ + GM	5.36ab	6.92	0.435	31.2	12.6	68.4
PTR_W25_ + GM	5.96a	6.98	0.459	33.2	12.6	70.2
LSD (*p* = *0.05*)	0.8	NS	NS	NS	NS	NS
**Wheat establishment methods**						
CTW_R0_	5.31ab	6.76b	0.44	32.6	12.6	68.9a
ZTW_R0_	4.81b	6.71b	0.42	29.1	12.4	63.9b
ZTW_R100_	5.77a	7.08a	0.45	33.8	12.7	70.5a
LSD (*p* = *0.05*)	0.54	0.22	NS	NS	NS	4.9
Interaction (A X B)	NS	NS	NS	NS	NS	NS

*LSD* Least significant difference, *NS* Non-significant. *PTR*_*W0*_ Puddled transplanted rice with no wheat straw, *PTR*_*W25*_ Puddled transplanted rice with 25% anchored wheat straw retained, *GM* Green manure, *CTW*_*R0*_ Conventional tillage wheat with rice straw removed, *ZTW*_*R0*_ Zero tillage wheat with rice straw removed, *ZTW*_*R100*_ ZTW with rice straw retained as surface mulch.

**Table 3:** Effect of tillage, green manure and residue management practices on phosphorus content and uptake in wheat after five years of rice–wheat cropping system.

Treatments	*P* in grain(%)	*P* in straw (%)	*P* uptake by grain	*P* uptake by straw	Total *P* uptake	PHI
			(Kg ha^-1^)			
**Rice establishment methods**						
PTR_Wo_						
PTR_W25_	0.31	0.027	15.7	1.86	17.5	0.89
PTR_W0_ + GM	0.3	0.033	16	2.28	18.3	0.97
PTR_W25_ + GM	0.28	0.034	16.7	2.39	19	0.87
LSD (*p* = *0.05*)	NS	NS	NS	NS	NS	NS
**Wheat establishment methods**						
CTW_R0_	0.28	0.03	14.8ab	2.07	16.9b	0.87
ZTW_R0_	0.29	0.029	13.8b	1.96	15.8b	0.87
ZTW_R100_	0.3	0.035	17.7a	2.49	20.2a	0.87
LSD (*p* = *0.05*)	NS	NS	3.2	NS	3.1	NS
Interaction (AX B)	NS	NS	NS	NS	NS	NS

*LSD* Least significant difference, *NS* Non-significant, *PTR*_*W0*_ Puddled transplanted rice with no wheat straw, *PTR*_*W25*_ Puddled transplanted rice with 25% anchored wheat straw retained, *GM* Green manure, *CTW*_*R0*_ Conventional tillage wheat with rice straw removed, *ZTW*_*R0*_ Zero tillage wheat with rice straw removed, *ZTW*_*R100*_ ZTW with rice straw retained as surface mulch.

**Table 4:** Pearson’s correlation coefficients (r) between phosphorus fractions, biological activity and crop yield.

Variable	WS-P	NaHCO_3_-Pi	NaHCO_3_-Po	NaOH-Pi	NaOH-Po	HCl-P	TP	Av-P	Alk-p	Phy-P	Yield
WS-P	1										
NaHCO_3_-Pi	0.992**	1									
NaHCO_3_-Po	0.954**	0.967**	1								
NaOH-Pi	0.868*	0.833*	0.865*	1							
NaOH-Po	0.930**	0.921**	0.959**	0.942**	1						
HCl-P	0.761*	0.752	0.718	0.835*	0.718	1					
TP	0.975**	0.966**	0.972**	0.943**	0.984**	0.793*	1				
Av-P	0.935**	0.944**	0.964**	0.842*	0.970**	0.608	0.957**	1			
Alk-P	0.979**	0.985**	0.932**	0.786*	0.904**	0.668	0.940**	0.950**	1		
Phy-P	0.972**	0.965**	0.909**	0.878**	0.881**	0.865*	0.950**	0.864*	0.929**	1	
Yield	0.972**	0.962**	0.963**	0.934**	0.988**	0.765*	0.997**	0.968**	0.949**	0.937**	1

*, **Significant at 0.05 and 0.01 probability level, respectively.

*WS-P* Water soluble phosphorus, *NaHCO*_*3*_*-P*_*i*_ Sodium bicarbonate inorganic phosphorus, *NaHCO*_*3*_*-P*_*o*_ Sodium bicarbonate organic phosphorus, *NaOH-P*_*i*_ Sodium hydroxide inorganic phosphorus, *NaOH-P*_*o*_ Sodium hydroxide organic phosphorus, *HCl-P* Hydrochloride phosphorus, *TP *Total Phosphorus, *Av-P* Available phosphorus, *Alk-P* Alkaline phosphatase activity, *Phy-P* Phytin-P.

### Principal component analysis

Principal component analysis was performed to extract the most influential soil parameters from each PC on the basis of eigen vector weight value or loading factors (Table 5). Only the highly weighted variables were retained in the minimum data set (MDS). The PC1 and PC2 explained 88.0% variability in the data set, where PC1 explained 74.68% and PC2 explains 13.32%. Hence, the bold-face values (NaOH-P_o_ and NaHCO_3_-P_i_ for PC1, HCl-P for PC2) were considered to be highly weighted eigen vectors and were initially selected in the MDS. The amount of variability explained by PC1 was 74.7%, with an eigen value of 7.47, which includes NaOH-P_o_, with the highest positive factor loading value (0.95), and NaHCO_3_-P_i_ (0.93) (Table 5). The component PC2 explained variance of about 13.3% and eigenvalue of 1.33 with the highest positive loading value for HCl-P with positive factor loading (0.78). Based on the percent variance to total variance, the weight of each PC ranged from 0.15 to 0.85.

**Table 5:** Loading values and percent contribution of assayed soil variables at surface soil layer on the axis identified by the principal component analysis.

Soil variables	PC1		PC2	
	Loading value	Contribution of variables (%)	Loading value	Contribution of variables (%)
WS-P	0.864	9.99	0.425	13.6
NaHCO_3_-P_i_	0.934	**11.7**	− 0.123	1.13
NaHCO_3_-P_o_	0.875	10.2	0.311	7.25
NaOH-P_i_	0.911	11.1	− 0.308	7.11
NaOH-P_o_	0.950	**12.1**	− 0.258	5.02
HCl-P	0.480	3.08	0.784	**46.1**
TP	0.794	8.44	0.192	2.75
Av-P	0.885	10.5	− 0.417	13.0
Alk-P	0.922	11.4	− 0.225	3.81
Phytin P	0.926	11.5	0.054	0.22
Eigenvalue		7.47		1.33
Variability (%)		74.7		13.3
Cumulative %		74.7		88.0
weight		0.85		0.15

*WS-P* Water soluble phosphorus, *NaHCO*_*3*_*-P*_*i*_ Sodium bicarbonate inorganic phosphorus, *NaHCO*_*3*_*-P*_*o*_ Sodium bicarbonate organic phosphorus, *NaOH-P*_*i*_ Sodium hydroxide inorganic phosphorus, *NaOH-P*_*o*_ Sodium hydroxide organic phosphorus, *HCl-P* Hydrochloride phosphorus, *TP* Total phosphorus, *Av-P* Available phosphorus, *Alk-P* Alkaline phosphatase.

**Figure 7:**
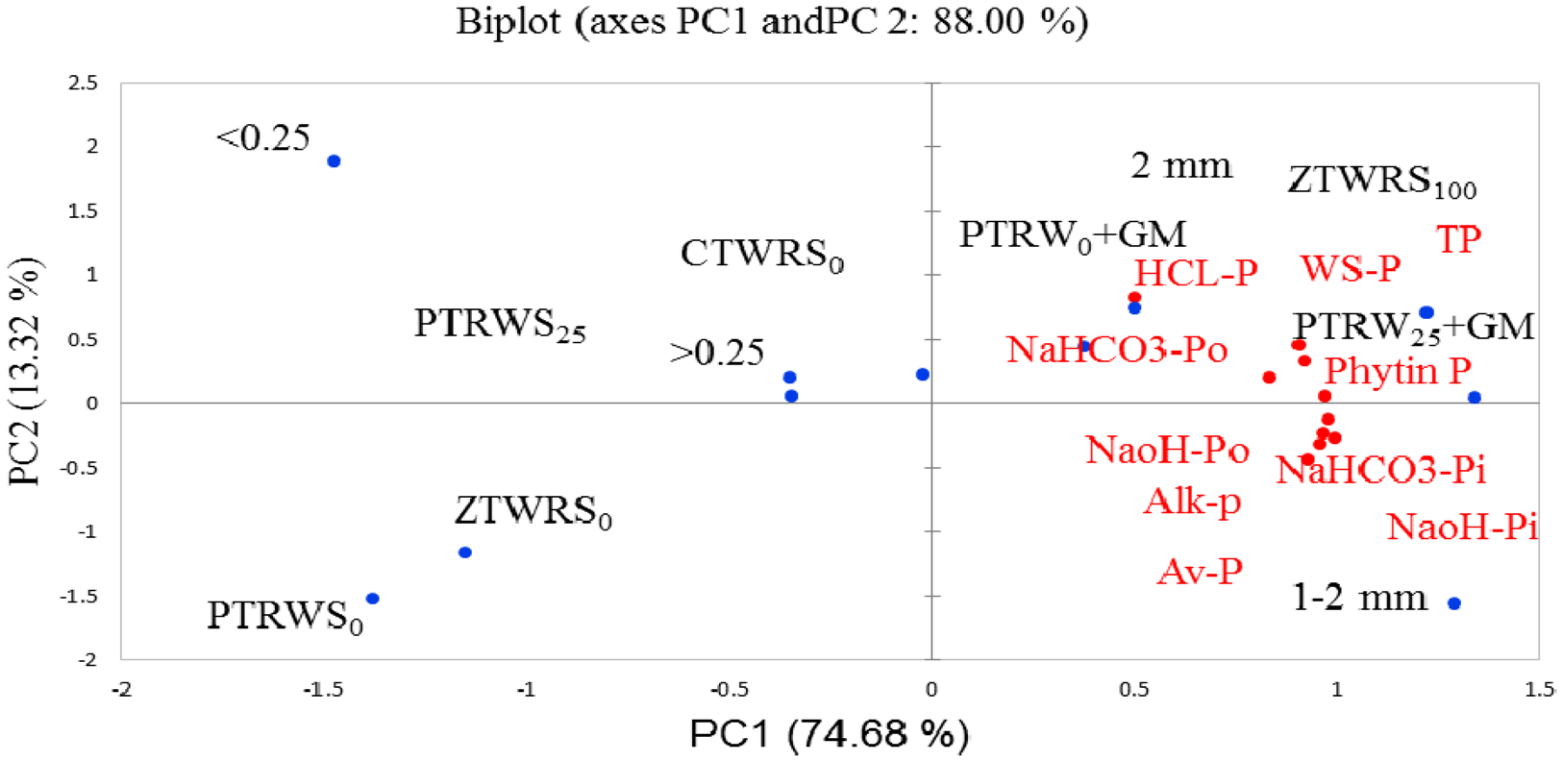
Principal component analysis (PCA) of assayed variables for studying the effect of tillage, crop residue management and green manure after five years of rice–wheat cropping system. PTR_W0_-puddled transplanted rice with no wheat straw, PTR_W25_-puddled transplanted rice with 25% anchored wheat straw retained, GM-Green manure, CTW_R0_-conventional tillage wheat with rice straw removed, ZTW_R0_-zero tillage wheat with rice straw removed, ZTW_R100_-ZTW with rice straw retained as surface mulch.. (Acronyms: WS-P Water soluble phosphorus, TP Total phosphorus, Av-P Available phosphorus, Alk-P Alkaline phosphatase).

**Figure 8:**
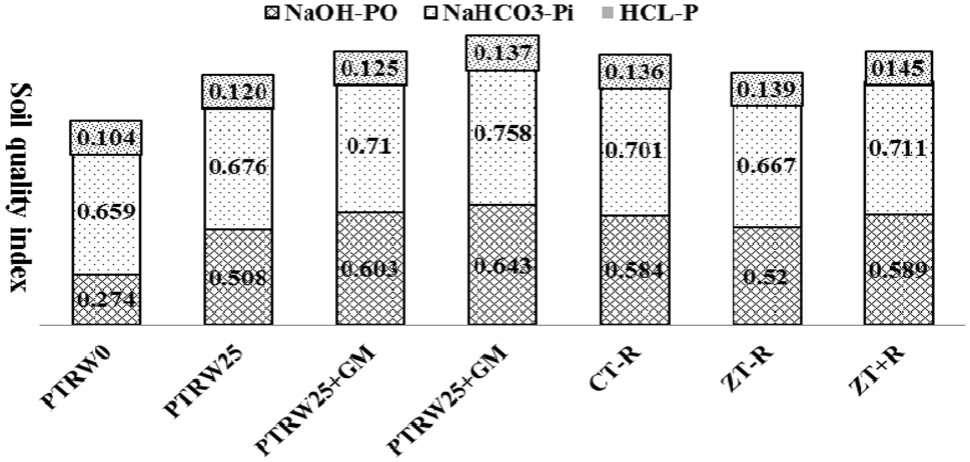
Average effect of rice and wheat treatments on soil quality index and the individual contribution of each of the key indicators. PTR_W0_-puddled transplanted rice with no wheat straw, PTR_W25_-puddled transplanted rice with 25% anchored wheat straw retained, GM-Green manure, CTW_R0_-conventional tillage wheat with rice straw removed, ZTW_R0_-zero tillage wheat with rice straw removed, ZTW_R100_-ZTW with 100% rice straw retained as surface mulch. (Acronyms: P_o_- Organic phosphorus, P_i_- inorganic phosphorus).

**Figure 9:**
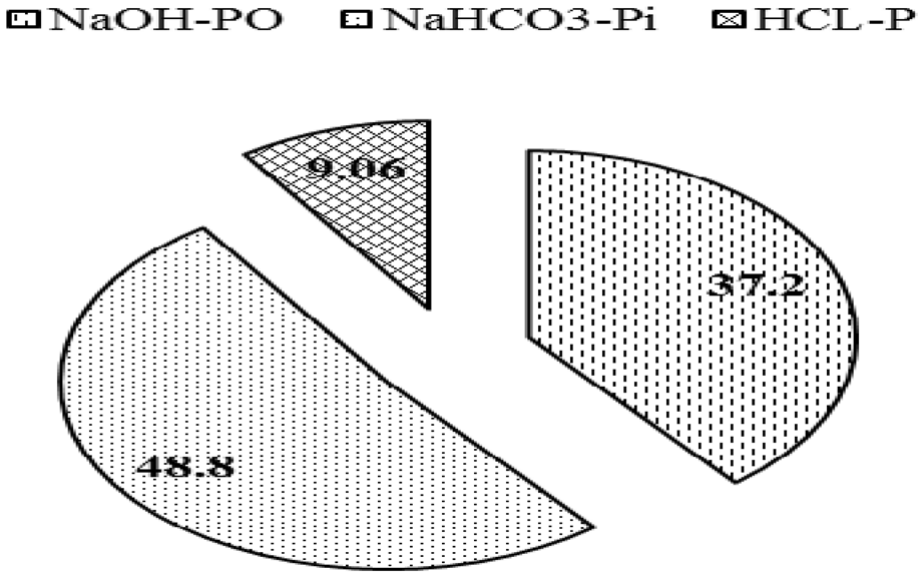
Overall contribution of the selected soil quality indicators to soil quality index.

**Figure 10:**
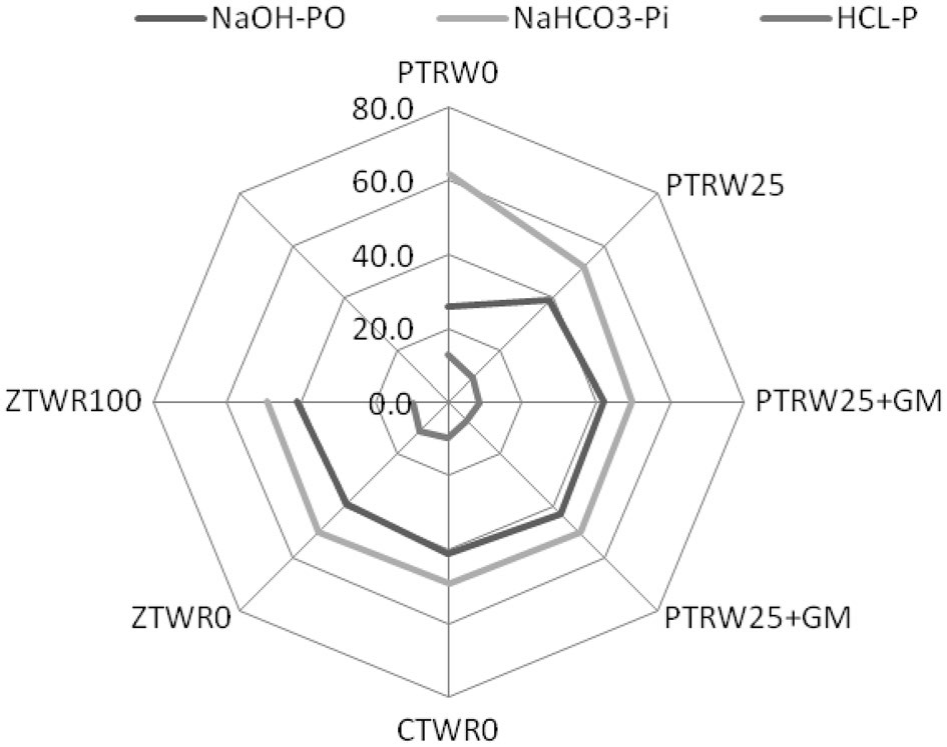
Contribution (%) of selected indicators to soil quality under rice and wheat treatments. PTR_WS0_-puddled transplanted rice with no wheat straw, PTR_W25_-puddled transplanted rice with 25% anchored wheat straw retained, GM-Green manure, CTW_R0_-conventional tillage wheat with rice straw removed, ZTW_R0_-zero tillage wheat with rice straw removed, ZTW_R100_-ZTW with 100% rice straw retained as surface mulch.

The position of different variables and treatments in the orthogonal space was defined by the two PCs (Fig. 7). The first principal component separated < 0.25 mm and > 0.25 mm size aggregates from 1–2 mm and > 2 mm size aggregates. It also clearly separated PTR_W25_ + GM and PTR_W0_ + GM from PTR_W0_ and PTR_W25_ treatment and ZTW_R100_ from ZTW_R0_ and CTW_R0_ treatments in the factorial space. The variables (NaOH-Po, NaHCO_3_-P_i_ and HCl-P) are related to the large size aggregates (both 1–2 mm and > 2 mm). These variables are also related to PTR_W25_ + GM, PTR_W0_ + GM and ZTW_R100_ treatments. The results displayed that the contribution of NaOH-P_o_ toward SQI was highest under PTR_W25_ + GM (0.643) followed by PTR_W25_ (0.508) in rice treatments and CTW_R0_ (0.584) followed by ZTW_R100_ (0.589) in wheat treatments (Fig. 8). For NaHCO_3_-P_i_ the maximum contribution toward SQI was observed under PTR_W25_ + GM (0.710) followed by PTR_W0_ (0.659) in rice treatments and ZTW_R100_ (0.711), followed by CTW_R0_ (0.701) in wheat treatments. The HCl-P contributed the maximum to SQI under PTR _W25_ + GM (0.137) in rice treatments and ZTW_R100_ (0.145) followed by CTW_R0_ (0.136) in wheat treatments. The relative order of contribution of the selected indicators to SQI was 37.2% for NaOH-P_o_, 48.8% for NaHCO_3_-P_i_, and 9.1% for HCl-P (Fig. 9). The radar plot represented the specific contribution of MDSs toward SQI. NaHCO_3_-P_i_ had the highest MSDs (49.3–61.6), HCl-P (6.9–12.8–16.4) had the lowest, whilst NaOH-P_o_ (25.6–42.7) was in-between for residue retention and green manure CA-based RWS (Fig. 10).

## Discussion

### Particle size phosphorus fractions in aggregates

The P added through residue plays an important role in regulating the mineralization or immobilization of soil P, thus altering the P transformations and affecting its availability^11,21^. Retention of crop residue promotes microbial activity, which enhances the aggregates cohesion and hydrophobicity^40^. Hangen et al.^41^ reported the destruction of soil macro-pores in conservation tillage and a further reduction in the loss of dissolved P through leaching. In the present study, the comparison of P-fractions among different aggregate size indicated that PTR_W25_ + GM and ZTW_R100_ recorded the highest P-forms under CA-based RWS. Also, the application of green manure and crop residue management practices has resulted in enhanced P content predominantly in aggregate size 1–2 mm relative to other sized aggregates. This may be because smaller aggregates possess a larger surface area, higher P sorption, and hence less P availability. Whereas, larger soil aggregates have less surface area, reduced P fixation and enhanced P availability^42^. As defined by Verma et al.^43^ and Chimdi et al.^44^, the water extractable and NaHCO_3_-P (P_i_ and P_o_) are considered as readily desorbable or labile phosphorus pools. This water soluble-P represents the readily available-P. The NaHCO_3_-Pi is the most biologically available inorganic P fraction and NaHCO_3_-Po is considered as the easily mineralized P fractions in the form of phospholipids and nucleic acid^45^. In our study, NaOH-P (P_i_ and P_o_) fractions were higher in treatment PTR_W25_ + GM and ZTW_R100_. The NaOH-P and HCl-P are sparingly labile-P and on a long-term basis, these forms might play an essential role in plant nutrition^46^. The present results indicated the dominance of HCl-P in all the aggregate size classes which is consistent with the results of Castillo and Wright^47^ who also observed the highest percentage of total P (41%) in Ca-bound P fraction (HCl-P) for sugarcane. Our findings also corroborated with the previous results by Ranatunga et al.^48^, who observed that long-term poultry litter application in pasture soil recorded the highest level of HCl-P in 1–2 mm macro-aggregate. The NaOH-P and HCl-P are sparingly labile-P and on long-term basis, these forms might play an essential role in plant nutrition. The RWS in the current study indicated the dominance of organic phosphorus fractions which may be due to the degradation of organic P and release of inorganic P for plants. The increase in P concentration under ZT is consistent with previous tillage studies by Essington and Howard^49^ who observed significantly higher organic P in ZT than CT. Our results also indicated that the wheat stubble and green manure in rice exhibited increased total and available P concentrations in the macro-aggregate fractions. This might be due to the long-term addition of crop residue to the soil, which increased the soil total phosphorus and available phosphorus contents^10^. The ZT and residue retention might have increased the labile P, P_o_ accumulation and its mineralization by phosphatases. A higher dissolved reactive P concentration in the leachate was reported by Gaynor and Findlay^50^ in ZT than CT. Previous studies have reported that ZT combined with straw retention, protects soil structure and aggregate-association and is an effective measure to improve soil structure, fertility and higher yields^3,23^.

### Soil alkaline phosphatase activity

Crop residue amended soils was more favorable for microbial growth that might have enhance the nutrient mobilization and inhibits the fixation of the available P by the soil^3,28^. The alterations in the soil biological dynamics could be easily revealed by the variations in enzyme activities^51^. The mineralization of P_o_ to available P_i_ is driven by phosphatases in the soil^52^ and play key roles in the soil system as a good indicator of soil fertility^53,54^. The long-term crop residue management practices caused a significant increase in microbial population and microbial biomass C or N in the soil^55,56^, thus providing energy and a favorable environment for the accumulation of soil enzymes^57^. Gupta and Germida^58^ observed that the macro-aggregates had higher phosphatase activity in crop residues retention and ZT than CT in their respective micro-aggregates, which corresponds with the present results. The higher aggregate associated inorganic P in our study may be due to higher microbial proliferation resulting from the retention of crop residues, the addition of GM and ultimately enhancing P_o_ mineralization over time. Similar findings were also mentioned by Margenot et al.^59^, who determined increased alkaline phosphatase activity (41%) under RT (reduced tillage) than CT. The highest activities of most of the enzymes under rice residue management practices in wheat are agreed with the earlier findings of Sharma et al.^3^. They observed that higher enzyme activities are associated with macro-aggregates than micro-aggregates due to improvement of organic carbon status of soils under rice residue retention in wheat. Higher phytin-P content in ZTW + R may be due to higher build up of organic matter^4,60^ that resulted in increased release of P from phytate present in the soil.

### Yield and nutrient uptake

The rice residue management practices are anticipated to have a positive impact on increasing P availability, which contributes towards increased crop yield^61^. Significantly higher grain yield in treatments PTR_W0_ + GM and PTR_W25_ + GM could be attributed to the addition of green manure which helps to enhance the availability of nutrients. Furthermore, the GM addition provides other essential nutritive substrates along with favorable conditions required for plants growth during the period of grain filling^62^. The higher grain yield increment with residue recycling (rice, wheat and GM) exhibited higher P acquisition capacity owing to their important functional traits like higher release of root exudates and deeper roots^18,63^. Similarly, the higher wheat yield was recorded under ZT with residue retention compared with CT. This enhanced yield might be related to the improvement in soil physical properties and water retention especially under ZT with residue retention than CT for nutrient accessibility^3^. The present results were consistent with the findings of Zhang et al.^63^ who reported slight increase in the yields of rape and rice by the NT (No-tillage) than CT across three years. Nandan et al.^64^ reported higher grain yield by the crop establishment practices based on ZT than CT in wheat and maize. The highest increase in productivity was recorded in maize (7–10%), followed by wheat (5–11%) and rice (3–8%) by retention of crop residues. The wheat grain yield was significantly higher by 7.3% and 17.5% in ZT with residue retention in comparison with CT and ZT with no residue, respectively. Also, the 11.5% higher productivity was recorded in puddled transplanted rice with wheat stubble + GM followed by ZT with residue retention compared with CT under RWS^27^. A significant increase in yield and macro-nutrients uptake in wheat and rice by tillage and rice-straw management practices were also reported by Sharma et al.^3^.

### Principal component analysis

The alterations in the soil properties were represented by the PCs with higher eigen values^65,66^ and for the interpretation, only PCs with eigen values > 1 were retained^67^. The data points for ZT have separated clearly from the data points for CT in the PCs defined factorial space (Fig. 8). The most influential variables for PC1 were NaOH-P_o_, NaHCO_3_-P_i_ and HCl-P for PC2 based on eigen vector weight value (Table 4). Thus, these parameters can be considered as potential indicators of P transformation under tillage, GM and residue management practices in RWS. These transformations act as a base for the decomposition of plants, aggregation in soil, soil tilth and availability of nutrients^68^. Among the PC1 indicators, NaOH-P_o_ accumulates more in the form of very stable organic compounds in soil than NaHCO_3_-Po such as inositol phosphates and their phytins^69,70^. In PC2, NaHCO_3_-P_i_ was found to have a significant correlation because NaHCO_3_-Pi is the most biologically available inorganic P fraction and NaHCO_3_-Po is considered as the easily mineralized P fractions in the form of phospholipids and nucleic acids^45^. In PC3, HCl-P was representative variable to benefit largely as Ca-P and residual-P as P in mineral matrices and very stable humic substances. The data point of the above variables are closely positioned to sustainable management practices (i.e. PTR_W25_ + GM and ZTW_R100_). This might be due to the positive effect of legume crops grown in RWS that has been reported to favor the net C build-up, associated aggregation and hydraulic properties^71^. Bera et al. ^18^ indicated a clear separation between ZT with crop residues and CT with no residue by PCA in wheat under RWS.

## Conclusions

This study concludes that tillage intensity, residue retention and green manure significantly enhance P fractions within aggregates compared with conventional tillage practices. The crop residues addition along with zero tillage preferred the higher amount of NaOH-P_o_, NaHCO_3_-P_i_ and HCl-P fractions in micro-aggregates than macro-aggregates and may act as dominant fractions in soil ensuring the P availability under RWS in sandy loam soils. The information on P fractions among different aggregates would be beneficial in the modification of current input management practices aimed for higher availability of P to plants along with sustained crop productivity. Therefore, tillage, green manure and residue management should be recommended and popularized for the sustainability of RWS.
